# Comparison of endoscopic submucosal dissection versus surgery for early gastric cancer in the elderly: a pooled analysis

**DOI:** 10.1186/s12957-023-03167-7

**Published:** 2023-09-07

**Authors:** Fei Liu, Ze-Lin Wen, Xu-Rui Liu, Zi-Wei Li, Quan Lv, Wei Zhang, Dong Peng

**Affiliations:** 1 Department of Gastrointestinal Surgery, the First Affiliated Hospital of Chongqing Medical University, Chongqing, 400016 China; 2 Department of Gastrointestinal Surgery, Chongqing Medical University, Yongchuan Hospital, Chongqing, 402160 China

**Keywords:** Early gastric cancer, Elderly patient, Endoscopic submucosal dissection, Overall survival

## Abstract

**Purpose:**

The aim of this study was to investigate whether there was a difference in overall survival (OS) between elderly patients with early gastric cancer (EGC) who underwent endoscopic submucosal dissection (ESD) and those who underwent surgery.

**Methods:**

Four databases including PubMed, Embase, the Cochrane Library and CKNI were searched on March 20, 2023. The characteristics of the studies and the baseline information of the patients, including their medical histories, postoperative data, and prognoses, were recorded. Odds ratios (ORs) or mean differences (MDs), and 95% confidence intervals (CIs) were pooled up to calculate baseline information and postoperative information. Hazard ratios (HRs) and 95% CIs were used to calculate the prognosis of the patients. Stata V16.0 software was used for the data analysis.

**Results:**

A total of eight studies involving 2334 patients were included for the data analysis in this study. After pooling up the data, we found that the ESD group had lower Eastern Cooperative Oncology Groupprevious (ECOG) scores (OR = 0.33, 95% CI = 0.17 to 0.65, I^2^ = 59.69%, *P* = 0.00 < 0.05) than the surgery group. There were significant differences in the operation time (MD = -3.38, 95% CI = -5.19 to -1.57, I^2^ = 98.31%, *P* = 0.00 < 0.05), length of hospital stay (MD = -3.01, 95% CI = -4.81 to -1.20, I^2^ = 98.83%, *P* = 0.00 < 0.05) and hospitalization expenses (MD = -2.67, 95% CI = -3.59 to -1.75, I^2^ = 93.21%, *P* = 0.00 < 0.05) between the two groups. The ESD group had a lower OS rate (HR = 2.81, 95% CI = 2.20 to 3.58, I^2^ = 12.28%, *P* = 0.00 < 0.05).

**Conclusion:**

Elderly patients with EGC who underwent ESD had a significantly worse OS rate than those who underwent surgery. If the patient’s condition was suitable, surgery was still recommended for these patients.

## Introduction

Gastric cancer (GC) is recognized as one of the most common malignancies in the world and is the third leading cause of cancer-related deaths [1–4]. In China, the incidence and mortality rates of GC are increasing, and approximately 400,000 new cases of GC are diagnosed each year [5]. At present, surgery is still the standard treatment [6–8].

As the proportion of elderly people increases and people take their health more seriously, the proportion of elderly people diagnosed with early gastric cancer (EGC) increases [9–11]. Elderly people are often in poor condition or have other comorbidities, and surgery may be overly invasive and may not improve the prognosis of elderly patients [12–14]. EGC is defined as cancer confined to the gastric mucosa or submucosa, regardless of lymph node metastasis. Therefore, the number of EGCs that are treated with endoscopic submucosal dissection (ESD) has increased [15].

ESD or surgical resection are selected as treatments for EGC. The effectiveness of treatment modalities (ESD or surgery) for elderly patients with EGC remains controversial. A study reported that ESD was associated with worse overall survival (OS) [16]. Other studies showed that there was no significant difference in OS between ESD and surgery [17–20]. Therefore, the aim of this study was to investigate whether there is a difference in OS between the ESD and surgery.

## Methods

Our pooling up analysis was produced in accordance with the Preferred Reporting Items for Systematic Reviews and Meta-Analyses (PRISMA) statement [21]. The registration ID of this study on PROSPERO is CRD42023445142, and the link is https://www.crd.york.ac.uk/prospero/display_record.php?ID=CRD42023445142.

### Search strategy

We searched four databases (PubMed, Embase, the Cochrane Library, and CNKI) on March 20, 2023. The key words of search strategy were ESD, elderly patients and GC. For ESD, we searched “endoscopic resection” OR “endoscopic submucosal dissection” OR “endoscopic mucosal resection”. For elderly patients, we searched “elderly patients” OR “older patients” OR “elderly people” OR “older people” OR elderly. In terms of GC, we searched “gastric cancer” OR “gastric carcinoma” OR “gastric neoplasms” OR “stomach cancer” OR “stomach carcinoma” OR “stomach neoplasms”. Each key word was made up of a topic word and free words. Between the topic words and the free words, “OR” was used. Then, the three key words were combined by “AND”. The searching fields were “title”, “abstract”, and “keywords”. Languages were limited to English and Chinese.

### Inclusion and exclusion criteria

The inclusion criteria of eligible studies were as follows: 1, all patients were diagnosed with EGC; 2, both the ESD group and the surgery group were reported; and 3, elderly patients were reported. The exclusion criteria were as follows: 1, case reports, case series, comments, letters to the editor, conference abstracts and nonoriginal articles; 2, data were repeated or overlapped; and 3, incomplete information.

### Study selection

Two authors searched the databases and identified eligible studies separately. First, duplicate studies were excluded. Then, the two authors scanned the titles and abstracts to find eligible studies. Finally, full text would be read to identify studies that could be included. Any disagreements were settled by a third author.

### Data collection

The information contained baseline characteristics of included studies and information of included patients. The studies’ characteristics included author, published year, country, study date, study type, sample size, language of the studies, and Newcastle–Ottawa Scale (NOS) score. As for patients’ information, age, sex, American Society of Anesthesiologists (ASA), Eastern Cooperative Oncology Groupprevious (ECOG), diabetes mellitus (DM), cardiovascular disease (CD), chronic kidney disease (CKD), liver dysfunction, tumor size, tumor location, histology, and invasion depth were collected. As for postoperative information, we included operation time, hospital stay, hospitalization expenses and fasting time. In terms of long-term outcome, OS was collected.

### Quality and evidence assessment

The ROBINS-I grade was used to evaluate the quality of the included studies [22]. The ROBINS-I scale contained 7 domains (bias due to confounding, bias in selection of participants into the study, bias in classification of interventions, bias due to deviations from intended interventions, bias due to missing data, bias in measurement of outcomes, bias in selection of the reported result). The levels of risk bias included low risk, moderate risk, serious risk and critical risk. GRADEpro (McMaster University, 2020, Ontario, Canada) was used to used for assessing the quality of the evidence.

### Statistical analysis

Dichotomous variables were described by odds ratios (ORs) and 95% confidence intervals (CIs). Mean differences (MDs) and 95% confidence intervals (CIs) were calculated for continuous variables. Hazard ratios (HRs) and 95% CIs were used to calculate OS of patients. To evaluate the statistical heterogeneity, the I^2^ value and the chi-squared test were used [23, 24]. We used the random effects model, and *P* < 0.05 was considered statistically significant [23]. Stata SE 16 was used for data analysis.

## Results

### Study selection

**Fig. 1:**
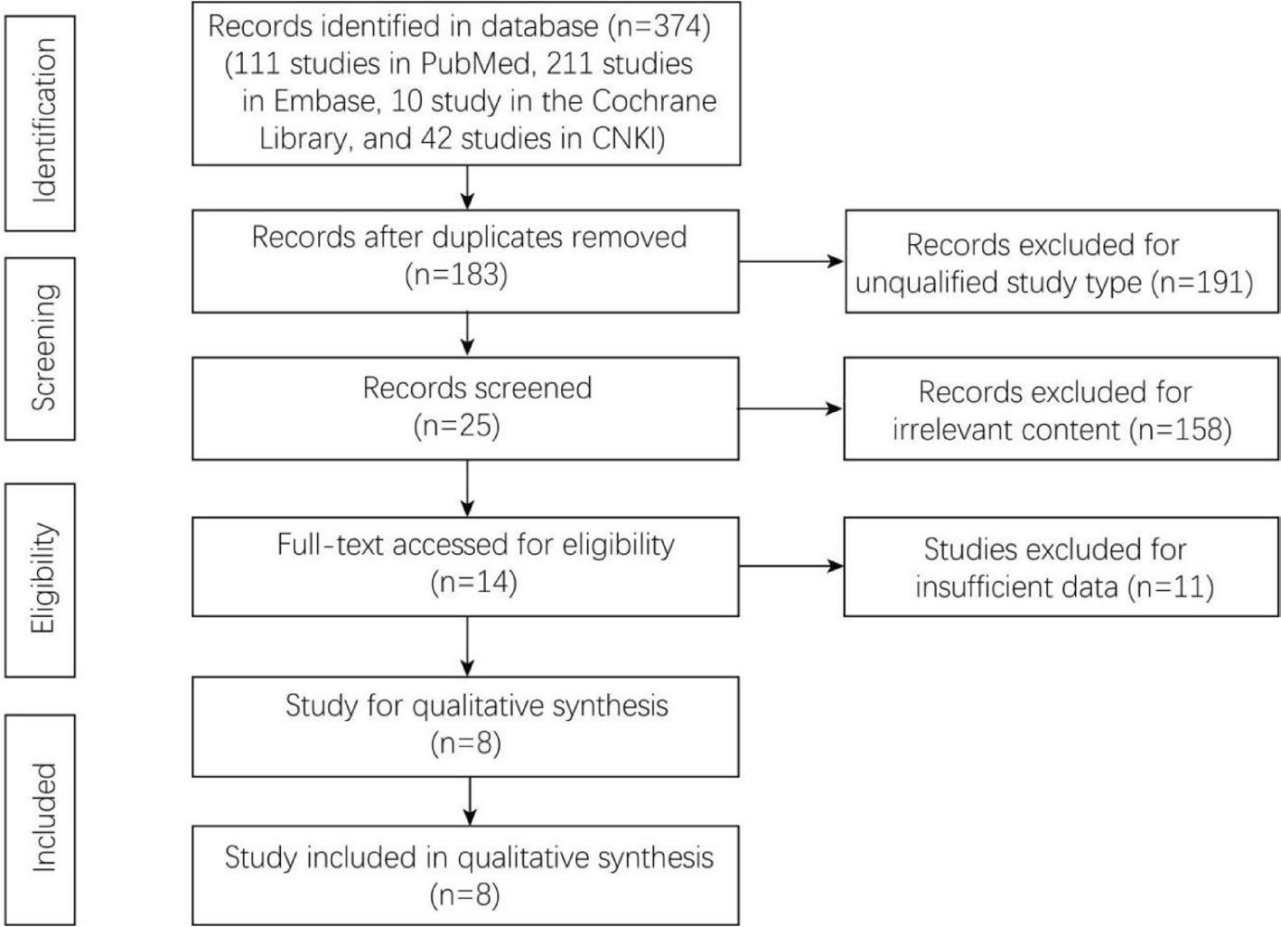
Flowchart of study selection

A total of 374 studies were searched from the three databases (111 studies from PubMed, 211 studies from Embase, 10 studies from the Cochrane Library, and 42 from CNKI). A total of 191 duplicate studies were eliminated. After the titles and abstracts of the remaining 183 studies were viewed, 14 studies were left for full-text screening. Eight studies were determined to be eligible and thereby included in this analysis [16–20, 25–27] (Fig. 1).

### Baseline characteristics of enrolled studies

A total of 2334 patients were included from the eight studies included in this study. All patients were divided into the ESD group and the surgery group (1017 in the ESD group and 1317 in the surgery group). All of the included patients were ≥ 60 years. The studies were mainly published between 2014 and 2023, except for one of them, which was published in 2005. The study period was from 1985 to 2018. Additional information (authors, published countries, study type, sample size, study language, ROBINS-I grade, and GRADE) is shown in Table 1.

**Table 1:** Baseline characteristics of included studies

Author	Year	Country	Study date	Patients	Age (year)	Study type	Sample size		Language	ROBINS-I grade	GRADEpro grade
							ESD	Surgery			
Kishida Y	2022	Japan	2012–2015	417	≥ 75	retrospective	114	303	English	Low Risk	Low
Park CH	2014	Korea	2007–2013	264	≥ 70	retrospective	132	132	English	Low Risk	Very low
Etoh T	2005	Japan	1985–1999	93	≥ 80	retrospective	49	44	English	Moderate Risk	Low
Kang S	2023	Korea	2005–2015	294	≥ 75	retrospective	59	235	English	Low Risk	Low
Miyahara K	2022	Japan	2006–2016	535	≥ 77	retrospective	365	170	English	Low Risk	Low
Jin Z	2021	China	2013–2018	487	≥ 60	retrospective	148	339	Chinese	Moderate Risk	Very low
Wen-Jin L	2020	China	2014–2016	140	≥ 60	retrospective	78	62	Chinese	Moderate Risk	Very low
Jiang T	2019	China	2013–2016	104	≥ 60	retrospective	72	32	Chinese	Moderate Risk	Very low

*Abbreviations*: *ESD* Endoscopic submucosal dissection

### Baseline characteristics of the patients

We pooled up the baseline characteristics of the included patients including age, sex, ASA, ECOG, hypertension, DM, CD, CKD, liver dysfunction, and tumor characteristics, including size, location, histology, invasion depth. After data analysis, we only found that the ESD group had a lower ECOG (OR = 0.33, 95% CI = 0.17 to 0.65, I^2^ = 59.69%, *P* = 0.00 < 0.05) than the surgery group. There were no significant differences in the other baseline information (*P* > 0.05). Additional details are shown in Table 2.

**Table 2:** Baseline information of the patients in the ESD and surgery groups

Characteristics	Studies	Participants (ESD/ Surgery)	Odds Ratio/Mean Difference (95% CI)	Heterogeneity
Age	8	1017/1317	0.24 [-0.06, 0.53]; *P* = 0.12	I^2^ = 90.26%; *P* = 0.00
Sex (male)	8	1017/1317	1.12 [0.93, 1.36]; *P* = 0.24	I^2^ = 0.00%; *P* = 0.97
ASA (≥ 3)	2	479/473	0.65 [0.27, 1.56]; *P* = 0.34	I^2^ = 44.81%; *P* = 0.18
ECOG (≥ 2)	2	479/473	0.33 [0.17, 0.65]; *P* = 0.00^*^	I^2^ = 59.69%; *P* = 0.12
Hypertension	3	352/503	0.77 [0.58, 1.03]; *P* = 0.08	I^2^ = 0.00%; *P* = 0.53
DM	4	401/547	0.79 [0.53, 1.18]; *P* = 0.25	I^2^ = 0.00%; *P* = 0.94
CD	4	401/547	0.66 [0.43, 1.01]; *P* = 0.06	I^2^ = 0.00%; *P* = 0.69
CKD	2	181/176	0.46 [0.18, 1.18]; *P* = 0.11	I^2^ = 0.00%; *P* = 0.91
Liver dysfunction	3	269/415	0.80 [0.35, 1.82]; *P* = 0.60	I^2^ = 0.00%; *P* = 0.58
Tumor size	7	885/1185	-0.93 [-1.92, 0.06]; *P* = 0.06	I^2^ = 98.82%; *P* = 0.00
Location	6			
Upper		187/293	1.03 [0.80, 1.33]; *P* = 0.80	I^2^ = 0.00%; *P* = 0.67
Middle		240/361	0.87 [0.66, 1.16]; *P* = 0.36	I^2^ = 26.58%; *P* = 0.24
Lower		468/589	reference	reference
Histology (diff/undiff)	8	1017/1317	1.92 [0.95, 3.86]; *P* = 0.07	I^2^ = 83.56%; *P* = 0.00
Invasion depth (M/SM)	5	753/920	2.85 [0.59, 13.75]; *P* = 0.19	I^2^ = 96.07%; *P* = 0.00

*Abbreviations*: *ESD* Endoscopic submucosal dissection, *ASA* American Society of Anesthesiologists, *ECOG* Eastern Cooperative Oncology Groupprevious, *DM* Diabetes mellitus, *CD* Cardiovascular disease, *CKD* Chronic kidney disease, *M* Mucosa, *SM* Submucosa

### Postoperative information

**Fig. 2:**
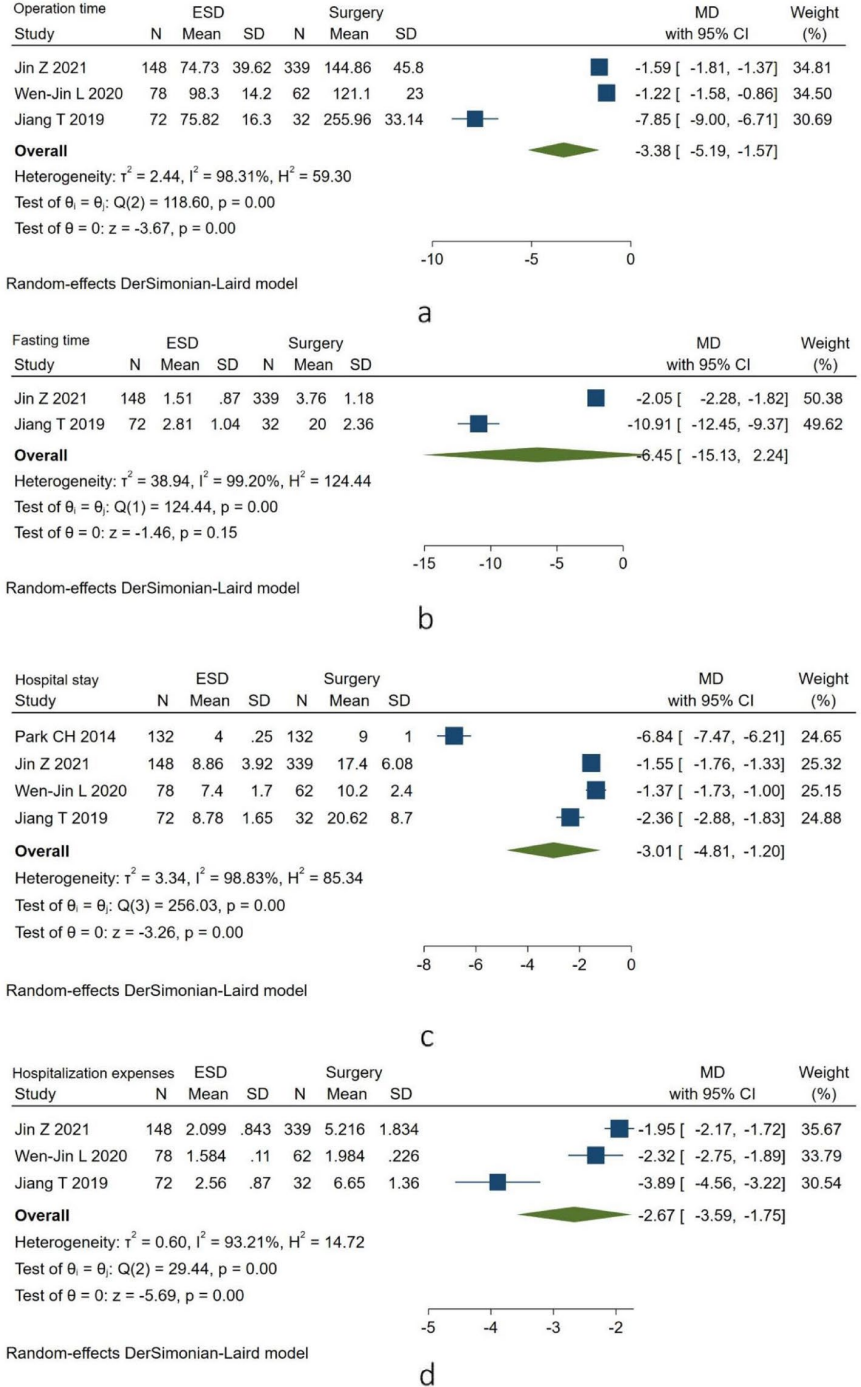
Postoperative information between the two groups. a, operation time between the ESD group and the surgery group; b, fasting time between the ESD group and the surgery group; c, hospital stay between the ESD group and the surgery group; d, hospitalization expenses between the ESD group and the surgery group. Abbreviations: ESD, endoscopic submucosal dissection

The operation time, fasting time, length of hospital stay, and hospitalization expenses were recorded. After the data analysis, there were significant differences in the operation time (MD = -3.38, 95% CI = -5.19 to -1.57, I^2^ = 98.31%, *P* = 0.00 < 0.05), length of hospital stay (MD = -3.01, 95% CI = -4.81 to -1.20, I^2^ = 98.83%, *P* = 0.00 < 0.05) and hospitalization expenses (MD = -2.67, 95% CI = -3.59 to -1.75, I^2^ = 93.21%, *P* = 0.00 < 0.05) between the two groups. However, the fasting time (MD = -6.45, 95% CI = -15.13 to 2.24, I^2^ = 99.20%, *P* = 0.15 > 0.05) was not significantly different between the two groups (Fig. 2).

### OS of the patients

**Fig. 3:**
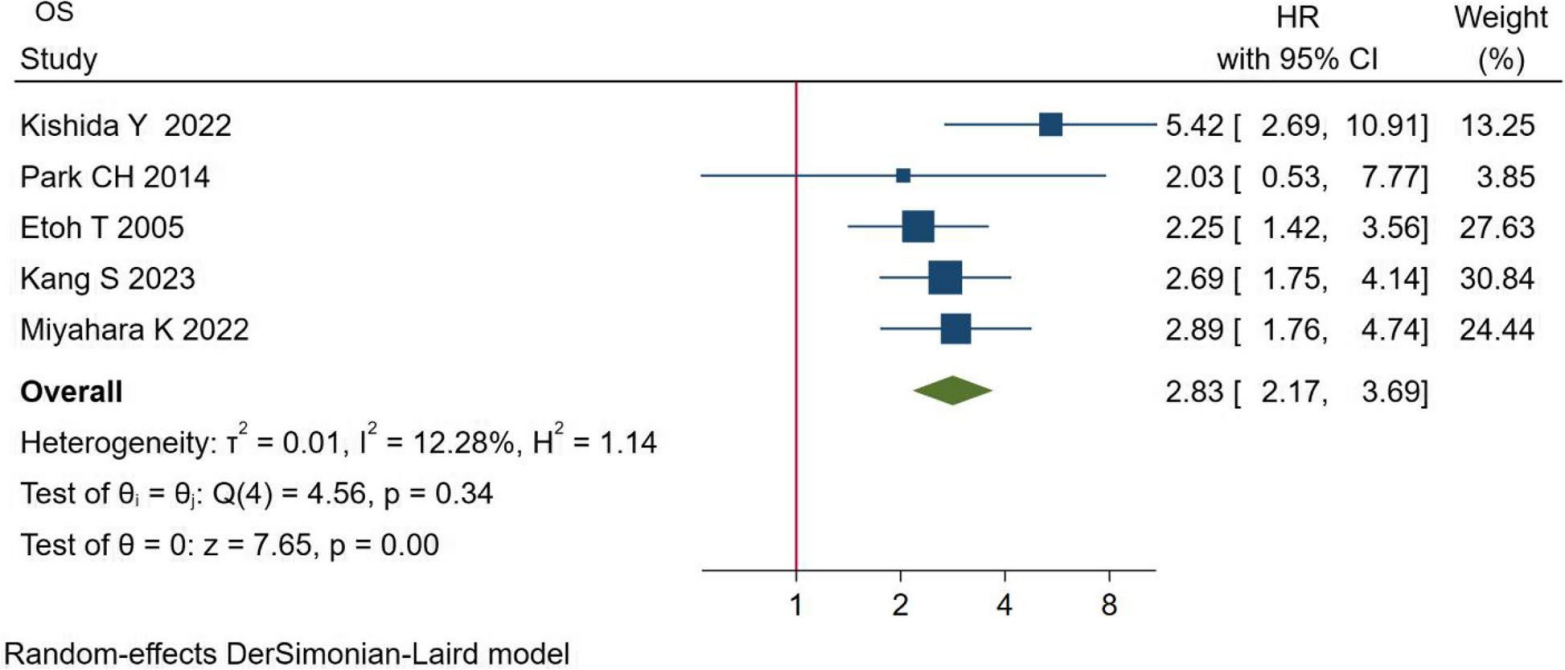
Comparison of OS between the two groups. Abbreviations: OS, overall survival

After pooling up the data, we found that the ESD group had a worse OS (HR = 2.81, 95% CI = 2.20 to 3.58, I^2^ = 12.28%, *P* = 0.00 < 0.05) (Fig. 3).

### Meta regression analysis

**Fig. 4:**
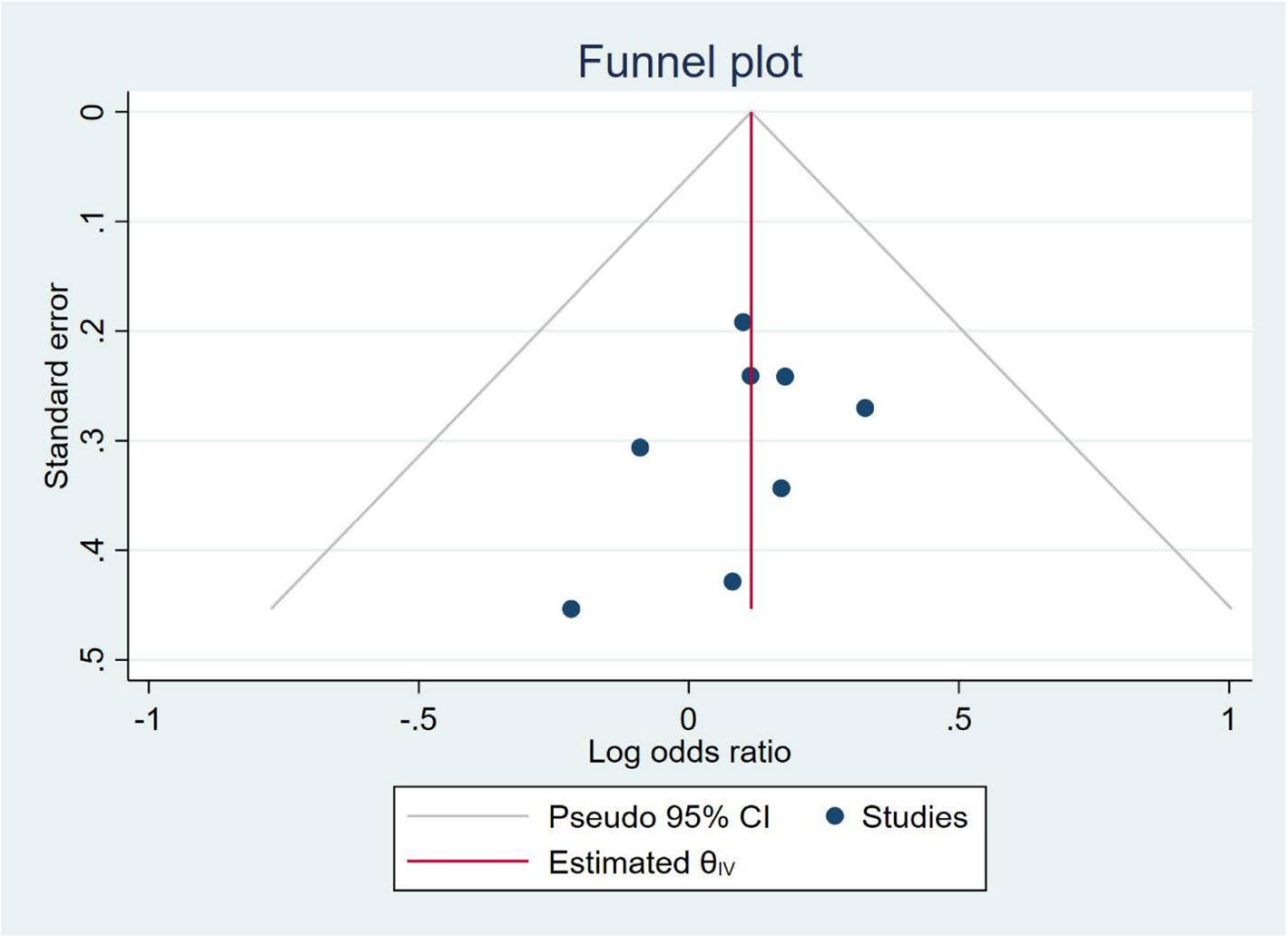
Funnel plot of overall complications

According to the data analysis, we did not find any source of heterogeneity (Fig. 4).

### Sensitivity analysis

The pooled analysis was repeatedly conducted to analyze the sensitivity by excluding each study, one at a time. The results were not significantly different after analyses after each exclusion.

## Discussion

A total of 2334 patients were included from the eight included studies in this study. According to the data analysis, the outcomes revealed that the ESD group had shorter operation time, shorter hospital stays and less hospitalization expenses. Moreover, the elderly EGC patients who underwent ESD had a worse OS than those who underwent surgery.

Surgical treatment remains the most effective means of curing EGC [28]. However, in elderly patients who often have other comorbidities, the prognosis is worse than that in younger patients, even though curative resection could be achieved with surgical treatment [13, 29, 30]. Due to the poorer conditions of elderly people, surgery might be too invasive and does not necessarily have a better prognosis. Compared to surgery, ESD is a minimally invasive treatment, and elderly patients can also be safely treated with ESD [31, 32]. ESD became more popular in elderly EGC patients. Many previous studies have compared the effects of ESD treatment with surgery in elderly patients with EGC. However, there is still no definitive difference between ESD treatment and surgical treatment for elderly patients with EGC. Some studies revealed that the OS between the ESD group and the surgery group was not significantly different [17–20]. Kishida Y et al. conducted a study of 417 elderly patients (114 in the ESD group; 303 in the surgery group) and found that the ESD group had worse OS than the surgery group [16]. Therefore, the aim of the current study was to investigate whether there was a difference in OS between the elderly patients with EGC who underwent ESD and those who underwent surgery.

The choice of treatment for elderly patients with EGC was not necessarily curative, and the patient's prognosis had to be fully considered. ESD, as a minimally invasive treatment for EGC, was proven to be a safe treatment [33, 34]. For the treatment of elderly EGC, ESD is gaining attention. However, the prognosis of ESD in elderly patients remains unclear. Some studies reported that the incidence of heterochronous lesions after ESD is higher than that after surgery [33, 35–37]. Moreover, EGC still carries a risk of lymph node metastasis, and ESD does not allow the removal of potentially metastatic lymph nodes [17–19]. According to the data analysis, we found that the elderly patients with EGC in the ESD group had a worse OS than those in the surgery group. The reason for this result was unclear, but the possible mechanism might be as follows: 1. patients in the ESD group might have lymph node metastases that could not be cleared by ESD; 2. OS might be impacted by differences in the baseline characteristics of the patients rather than by differences in the treatment effect, since this population had a high rate of deaths from other causes.

On the other hand, Etoh T et al. showed that for elderly patients with EGC, surgery could be performed safely [18]. Cheng YX et al. [38] reported that age might not have been an independent prognostic factor affecting OS in patients with GC who underwent gastrectomy. However, we found that the OS in the ESD group was worse. In terms of OS, surgery might be a better choice for elderly patients with EGC. Moreover, we found that the ESD group had shorter operation time, shorter hospital stays and less hospitalization expenses than the surgery group. For these factors, ESD might be a better choice than surgery. Therefore, for elderly EGC patients in poor condition and with high anesthesia risks, surgery might not be the best treatment option, and ESD might be an acceptable treatment instead.

To our knowledge, this study was the first study to pool the comparative prognosis in GC patients who underwent ESD or surgery in previous studies. However, there were some limitations of this pooled analysis. First, there were inconsistent inclusion criteria, some elderly patients were ≥ 70 years of age, and some were ≥ 80 years of age. Second, all the studies were conducted in East Asia, which might have caused selection bias. Third, there was no consideration of the effect of other factors on OS. Forth, we could only extract the OR to evaluate dichotomous variables. Therefore, more detailed research on this topic is needed in the future.

In conclusion, elderly patients with EGC who underwent ESD had a worse OS than those who underwent surgery. If the patient’s condition was suitable, surgery was recommended for these patients.

## Data Availability

The datasets used and analyzed during the current study are available from the corresponding author on reasonable request.
